# Targeted Treatment of Schizophrenia Symptoms as They Manifest, or Continuous Treatment to Reduce the Risk of Psychosis Recurrence

**DOI:** 10.1093/schbul/sbad145

**Published:** 2023-10-31

**Authors:** Michael Davidson, William T Carpenter

**Affiliations:** Department of Basic and Clinical Sciences, Psychiatry, University of Nicosia Medical School, 2414, Nicosia, Cyprus and Minerva Neurosciences, 1500 District Avenue, Burlington, MA 01803, USA; University of Maryland School of Medicine, Department of Psychiatry, Maryland Psychiatric Research Center, Baltimore, MD, USA

**Keywords:** treatment, antipsychotics, psychosis, targeted, symptoms, continuous, adverse effects, discontinuation

## Abstract

Current pharmacological treatment of schizophrenia employs drugs that interfere with dopamine neurotransmission, aiming to suppress acute exacerbation of psychosis and maintenance treatment to reduce the risk of psychosis recurrence. According to this treatment scheme, available psychotropic drugs intended to treat negative symptoms, cognitive impairment, or anxiety are administered as add-ons to treatment with antipsychotics. However, an *alternative* treatment scheme proposes a targeted or intermittent treatment approach, by which antipsychotic drugs are administered upon psychosis exacerbation and discontinued upon remission or stabilization, while negative symptoms, cognitive impairment, or anxiety are treated with specific psychotropics as monotherapy. Along these lines, antipsychotics are renewed only in the event of recurrence of psychotic symptoms. This 50-year-old debate between targeted and continuous treatment schemes arises from disagreements about interpreting scientific evidence and discordant views regarding benefit/risk assessment. Among the debate’s questions are: (1) what is the percentage of individuals who can maintain stability without antipsychotic maintenance treatment, and what is the percentage of those who exacerbate despite antipsychotic treatment? (2) how to interpret results of placebo-controlled 9- to 18-month-long maintenance trials in a life-long chronic disorder, and how to interpret results of the targeted trials, some of which are open label or not randomized; (3) how to weigh the decreased risk for psychotic recurrence vs the almost certainty of adverse effects on patient’s quality of life. Patients’ profiles, preferences, and circumstances of the care provision should be considered as the targeted vs continuous treatment options are considered.

## Background

Serendipitous observations and pharmacological inferences established between the 1950s and the 1970s that drugs that interfere with dopamine (DA) neurotransmission, ameliorate agitation, delusion, hallucination, and thought disorder in patients suffering from schizophrenia.^1^ Since agitation is the first and most profoundly impacted symptom, the drugs were initially named “major tranquilizers” to be distinguished from benzodiazepines called “minor tranquilizers.” Because the natural course of schizophrenia is characterized by alternating periods of psychotic (positive) symptom exacerbation, improvement, and recurrence of psychosis, researchers since the 1970s have investigated the possibility that the same DA-blocking drugs might also reduce the risk of psychotic symptoms re-emergence.^2^ The fact that the same drug that might ameliorate symptoms during a disease flare-up can also reduce the risk of future recurrences is well documented in medicine: for instance, steroids by changing the inner functioning of the cell, ameliorate flare-ups of rheumatoid arthritis, allergic conjunctivitis, or asthma and reduce the risk of future flare-ups. Like antipsychotics in schizophrenia, the benefit/risk of intermittent vs continuous treatment with steroids is still debated, many decades after steroids has been available in clinical practice.^3,4^

Based on randomized controlled trials (RCTs), most treatment guidelines issued by professional organizations recommend that individuals who meet diagnostic criteria for schizophrenia and experience recurrences of several psychotic episodes should continue antipsychotic treatment for many years to indefinitely.^5–7^ However, these guidelines are actively debated,^8–13^ and the more recent clinical guidelines tend to include intermittent treatment as an option.^14^ They propose a targeted therapy approach by which DA-blocking antipsychotic drugs are discontinued upon psychotic symptoms’ remission or stabilization, to be renewed only in case of recurrence of psychosis.

The case for continuous maintenance treatment is straightforward in that for many patients, the risk of symptoms re-emergence persists long after the resolution of the acute symptoms.^15,16^ The recurrent but debated argument is that the short- and long-term impact of psychosis exacerbations and hospitalizations on the patient’s well-being justifies the immediate and cumulative adverse effects of the drugs.^17^ Furthermore, as indicated by some,^18^ but not others,^19^ relapses might negatively impact on the long-term outcome as symptoms may not return to the pre-discontinuation baseline. Insomnia, anxiety, and brief and transient psychotic ideations might herald psychotic exacerbation, yet the criteria that distinguish between individuals who can maintain the stability of psychotic symptoms in the absence of antipsychotics and those who would experience rapid exacerbation are not validated predictive tests.^20^ Therefore, all individuals affected by chronic schizophrenia and recurrences of psychotic illness should be encouraged to accept continuous, long-term maintenance treatment.

The case for the targeted or intermittent treatment argues that many patients who meet phenomenological criteria for schizophrenia may not suffer from a DA-related abnormality hence, treatment with drugs affecting DA neurotransmission should be viewed as a symptomatic treatment rather than as a long-term treatment or as a drug-symptom interaction rather than a drug-disease interaction.^21^ In fact, studies that compared continuous maintenance treatment and treatment discontinuation after the resolution of the acute episode produced mixed results. Targeted/intermittent treatment was associated with more incidents of psychotic symptoms relapse compared to the maintenance group,^22,23^ but there were no advantages in terms of social and vocational outcomes for the maintenance group.^24^ Like almost all pharmacological interventions, antipsychotic treatment has immediate and cumulative adverse effects; therefore, its use should be restricted to the minimum regarding dose and duration. Moreover, several follow-up studies have indicated that schizophrenic patients on intermittent treatment have better long-term social and vocational performance outcomes than those on continuous treatment.^8,25–29^

## Interpretation of Scientific Evidence

### Methodological Limitations

RCTs comparing maintenance treatment to placebo use events such as violent outbursts or hospitalizations, as surrogate outcomes for illness worsening and exacerbation in addition to changes on psychometric scales. However, such outcomes could occasionally reflect the interpretation of odd behavior in an individual diagnosed with schizophrenia rather than true relapse of psychosis.^12^ Furthermore, questions have been raised whether changes on psychometric scales, while being sufficient to demonstrate statistically significant differences between drug and placebo, are also clinically meaningful to reflect illness exacerbation.^30,31^ Also, maintenance trials comparing a drug that interferes with DA neurotransmission to placebo cannot be genuinely blinded since the drugs have Adverse Effects (AE) that are known and anticipated by both patient and the investigator/rater, which biases the results in favor of the active drug over placebo.^32,33^ Howevere, a registry-based study that reported shorter intervals between hospital admissions in patients who discontinued antipsychotics compared with the maintenance group,^34^ like most registry-based studies, could not control for relevant confounders like distinguishing between true adhering to the treatment as prescribed or just medication retrieval from the pharmacy.^33,35^

Also, placebo-controlled trials supporting maintenance treatment last 6–12 months, which might be too short to be informative for an illness with a lifetime course.^36,37^ Supporting this assertion is a trial showing that psychosis was less likely to worsen in patients allocated to continued maintenance treatment in the short run, but in the long run, social functioning was worsened by maintenance treatment.^26^ Finally, discontinuation of antipsychotics might produce withdrawal effects beyond supersensitivity (see infra supersensitivity) such as insomnia which by itself might contribute to psychosis exacerbation. Moreover, patients released from the sedating effects of antipsychotics might appear to the untrained eye as agitated hence, misdiagnosed as exacerbated.^38–40^

Unlike RCT, intermittent/targeted treatment studies typically last several years. However, despite efforts to design the most valid studies within the constraints of very long-term follow-up and significant drop-out rate,^41^ such studies have inevitable methodologic weaknesses.^42^ Because some of the follow-up studies did not use a random assignment design,^24^ the possibility that the results are confounded by indication cannot be ruled out. It is possible that less severely ill patients at baseline were included in the intermittent or no treatment group, raising the *chicken and egg* question or inverse causality concerns.^43,44^ However, even if that has been the case, it still demonstrates that a subgroup of about 30% of patients diagnosed with schizophrenia does not experience psychotic symptoms in the absence of antipsychotic drugs.^29^ Furthermore, at least 1 study demonstrating the advantages of targeted therapy used a random assignment design.^26^

Also, it is possible that the percentage of patients who live in the community without antipsychotics maintenance treatment is higher than the 30% of patients who do not manifest psychotic exacerbation in the RCT when randomized to placebo. Patients who participate in RCT trials or are treated in clinical practice are generally those who experience active and recurrent psychotic symptoms, while those who experience only residual, mostly negative symptoms tend to be lost to psychiatric follow-up despite poor social and vocational functioning.

### Brain Volume Loss

Meta-analyses of imaging studies have demonstrated a correlation between cumulative exposure to antipsychotics and brain tissue loss,^45–48^ which might be responsible at least partialy for the cognitive^49,50^ and social^51^ impairments. Yet, limited brain atrophy has been reported in premorbid and first-episode patients who were drug naive or have received antipsychotics only for brief periods,^52^ suggesting that larger cumulative doses of antipsychotics could reflect a more severe form of illness, which could account for the brain atrophy and not the antipsychotic treatment. Also, examination of brain tissue from cognitively impaired schizophrenia patients exposed to very large doses of antipsychotics for several decades did not reveal any specific brain lesion or severe atrophy.^53^ Regardless of the reason for brain tissue atrophy or the presence or absence of brain lesion(s), like all biological findings in schizophrenia, brain volume loss reflects a group effect rather than a diagnostic biological marker on a known pathophysiologic path. Therefore, its contribution to the illness manifestation, if any, remains unknown.

### Metabolic Abnormalities and Cardiovascular Morbidity/Mortality

It is beyond debate that maintenance treatment with antipsychotic drugs increases the risk of diabetes, abnormal blood lipids, weight gain, and cardiovascular death.^54,55^ On the other hand, data indicate that compliance with antipsychotic medication might reduce death rate.^34^ One explanation for such discrepancy is that the reduced rate of death associated with compliance with antipsychotic treatment reflects a general tendency to be compliant with medical treatment rather than a direct benefit of antipsychotic drugs and that premature death reflects poor access to medical care and suicide, rather than the damage of cumulative treatment with antipsychotics. Furthermore, like most registry based studies the study associating reduced mortality with antipsychotic treatment^34^ suffers from unavoidable methodologic limitations.^56^

### Limited Effectiveness of Antipsychotic Drugs

Although the introduction, on a large scale, of antipsychotics coincides with the movement of deinstitutionalization, their contribution to deinstitutionalization is questionable.^57^ Drugs that directly interfere with DA neurotransmission have a relatively small effect size,^58^ are effective mainly for acutely agitated behavior and some aspects of psychosis, while the phenomenology of schizophrenia includes persistent negative symptoms, impairment in judgment, and cognitive and social functioning impairments on which antipsychotics have no direct therapeutic effect.^59–61^ Furthermore, it is not obvious whether maintenance treatment has a real impact on the social and vocational reintegration of patients, most of whom remain socially isolated and unemployed.^62^ Moreover, the beneficial effects of antipsychotics seem to decrease as a function of study duration possibly due to a tolerance effect to antipsychotics.^17,63^ However, proponents of the continuous treatment hypothesized that the biological effect of the drugs remains unchanged, but as the trial continues, compliance with treatment decreases, and trial participants drop-out, nullifying the advantage of being in the active arm vs the placebo.^7,64^

### DA Receptors Supersensitization

Continuing blockage of DA receptors by long-term maintenance treatment may cause receptor supersensitivity, which might account for the considerable proportion of patients in whom antipsychotics fail to produce improvements^65,66^ and which upon abrupt discontinuation, contribute to psychosis recurrence.^67^ This idea is supported by studies in rodents demonstrating that chronic treatment with antipsychotics increases DA receptor densities,^68^ and by some human studies that recommend gradual rather than abrupt discontinuation.^39,69^ An alternative explanation for rapid worsening upon drug discontinuation is the possibility that antipsychotic drugs with intrinsic anticholinergic effects produce cholinergic rebound upon discontinuation, which manifests as general malaise and can be mistaken for symptom worsening.^70^ Of note, a meta-analysis that looked at trials comparing abrupt vs gradual discontinuation of DA-blocking drugs found no differences between abrupt and gradual discontinuation regarding symptom worsening.^17^ However, it is possible that the rate of discontinuation was too fast to address the supersensitivity issue. Some^34^ but not all^71^ publications support this assertion, showing that most relapses occur many months and years after discontinuation rather that shortly after discontinuation.

## The Different Points of View in Benefit/Risk Assessment

Most decisions in daily life, including crossing the street, result from benefit/risk assessment, which is particularly true for the practice of medicine.^72^ Although all stakeholders of medical care, and the patients themselves, have the patient’s best interest as their primary purpose, the information available to each stakeholder to weigh benefit vs risk differs.

### Patients

Acute manifestation of psychosis is associated with severe patient suffering, active intervention of mental health professionals, and, very often, hospitalization. Under these circumstances, patients are willing and, under extreme circumstances, compelled to accept treatment with antipsychotic medications. However, when it comes to maintenance treatment as outpatients, particularly when psychosis is in remission, up to 70% of the patients discontinue medication on their own or do not adhere to it as prescribed.^73^ Surprisingly, the specific antipsychotic or the formulation (depot vs oral) does not seem to increase treatment adherence or improve outcomes.^74^ On the contrary, it is possible that irregular rather than continuous medication presents some advantages.^75^ However, as indicated by the proponents of depot medication, it is also conceivable that treatment-adherent patients are more likely to participate in prospective comparison trials of oral vs depot medication, thus obscuring the advantages of depot medication.^76^

For patients who are first-hand experts at AE, the wish to avoid akathisia, rigidity, tremor, sedation, lethargy, slowness of thinking and movements, and weight gain,^77^ which affect daily life, might prevail over the concern regarding increased risk of hospitalization.^10,78–80^ Furthermore, since some AE such as Parkinsonism are visible to others, it might amplify the stigma associated with the illness.

### Mental Health Professionals

Mental health professionals, having seen repeated psychotic exacerbations in patients who discontinue medications—patients’ anguish, family frustration, repeated hospitalization, self-defeating behaviors with life-disruptive consequences, and occasionally violence—tend to advise patients to tolerate continuous maintenance treatment despite the adverse effects. Although results of RCT consistent with such advice are the foundation of clinical decision-making, decisions of mental health professionals are also affected by psychological biases. The recent memory of the last patient who discontinued treatment, exacerbated, and “got in trouble” (ie, availability bias),^81^ probably affects treatment decisions more than all the patients who were lost to follow-up because they have left the care system and were living in the community without active symptoms or treatment. Also, a prescribing psychiatrist might be inclined to feel morally and at times legally, responsible for concurring with the patient’s wish to discontinue medication, which subsequently exacerbates and “got in troubles,” but not for the unavoidable drugs AE. Also, negative outcomes, such as risk of psychosis exacerbation, hospitalization, or aberrant behavior, are given more weight in medical decisions than positive outcomes, such as the certainty of living without the AE of drugs (ie, negativity bias).^82^

Since impaired judgment is common in many individuals suffering from schizophrenia, the patients’ negative attitude towards medication is often attributed to poor insight into the illness and misjudgment of the benefits of medication.^83,84^ However, this view, although not devoid of scientific support,^85^ aligns poorly with notions of respect for patients’ autonomy^86^ and shared decision-making.^87^

### General Public

While the risk of violent behavior in schizophrenia is probably exaggerated,^88^ the possibility that antipsychotic treatment might reduce the risk of violence^89^ makes mental health professionals and administrators, who see themselves as morally and legally responsible for the safety of the patients and their surroundings, disinclined to consider intermittent treatment.

Also, in an environment of limited resources, intermittent treatment requires more frequent interactions between patient and care provider than is necessary for continuous maintenance treatment. This is because antipsychotic discontinuation is probably safer if done gradually over several visits.^38^ Once the patient is drug-free, frequent contact (face to face or remote) is advisable to detect impending symptoms worsening, ie, anxiety, insomnia, social withdrawal,^90–94^ and to treat the symptoms before the full-blown exacerbation of psychosis. Remote patient monitoring of general activity, voice, movements, and social interactions are some of the newer methods that could alert the care provider of impending worsening.^95^

## Treating Symptoms Rather Than the Schizophrenia Syndrome

The diagnostic concept of schizophrenia syndrome encompasses individuals who differ widely in terms of phenomenology, illness course, and probably biological abnormalities.^96,97^ The Krepelanian dogma of a single outcome of life-long inevitable deterioration has been challenged by accumulating evidence.^98^^,99^ Schizophrenia heterogeneity is supported by the Genome-Wide Association Study (GWAS) studies indicating that many genetic variations can produce clinical phenomena that meet diagnostic criteria for schizophrenia.^100^ Also, studies that report many plausible biological abnormalities differences between cohorts of schizophrenia patients and controls are too often based on outliers of a few members of the patients’ cohort, further rejecting a unitary biological basis for this condition.

The quest for a unitary etiology or a repetition of the spirochete insanity solution for schizophrenia,^101^ in which a single abnormality/lesion is at the root of the 3 main component manifestations—psychosis, negative symptoms, and cognitive deficiency—would be convenient from the treatment point of view, but is not supported by evidence. On the contrary, it can be hypothesized that, at least in some patients, the 3 symptom clusters might be unrelated etiologically and manifest only by chance in the same individual who meets the criteria for schizophrenia. Psychosis ranges from a single episode to treatment-refractory,^102^ cognitive performance scores range from 2-SD below norms to occasionally above average performance scores,^103,104^ and negative symptoms from absent to life-long persistent and totally disabling. Also, the 3 symptoms are present trans-diagnostically. A certain degree of cognitive impairment,^105^ negative symptoms, and psychosis are present in many Central Nervouse System (CNS) disorders.

Taken together, evidence supports the idea of symptom-specific treatment as it manifests rather than a “one measure fits all,” as the current practice for chronic schizophrenia treatment. Despite the quest to reduce the risk of psychotic recurrence and treat primary negative symptoms and/or cognitive impairment, by adding a pro-cognitive or anti-negative symptom drug to an antipsychotic drug this strategy may not achieve the goal. Dozens of pharmaceutical industry-sponsored trials and hundreds of investigator-initiated ones in which psychotropics were added to an antipsychotic to treat specific symptoms, such as negative symptoms or cognitive impairment, have either failed, could or could not replicate the initial positive result^64^ or reported positive but post hoc findings.^106^ This might occur because maintenance with antipsychotics might produce secondary negative symptoms^107^ on top of the intrinsic primary schizophrenia-related negative symptoms. Since DA neurotransmission mediates brain reward circuits,^108–110^ it can be hypothesized that blocking DA receptors might deprive patients of experiencing pleasure and expecting reward(s), hence accounting for avolition, a widespread symptom of schizophrenia.^107,111^ This, in turn, lets a panel of investigators to suggest that the design of trials targeting negative symptoms should be monotherapy rather than an add-on to an antipsychotic drug.^112^

In summary, between patients who undergo good recovery from an episode of psychosis,^113^ those who can maintain long periods of symptom stability in the absence of maintenance treatment, and those who do not respond to treatment,^114,115^ it is possible that up to half of the individuals on maintenance therapy are exposed to the adverse effects of antipsychotics with only marginal benefit. Two ongoing large randomized, open-label trials comparing intermittent to continuous treatments,^116,117^ should provide needed evidence to this debate. The trials collect demographic, phenomenological, and biologic data intended to distinguish between patients who can and those who cannot maintain a long period of symptom stability without continuous maintenance treatment. Until this evidence is available, mental health professionals should utilize the imperfect yet available evidence to discuss with patients both treatment options.
